# What Is the Relationship between Outdoor Time and Physical Activity, Sedentary Behaviour, and Physical Fitness in Children? A Systematic Review

**DOI:** 10.3390/ijerph120606455

**Published:** 2015-06-08

**Authors:** Casey Gray, Rebecca Gibbons, Richard Larouche, Ellen Beate Hansen Sandseter, Adam Bienenstock, Mariana Brussoni, Guylaine Chabot, Susan Herrington, Ian Janssen, William Pickett, Marlene Power, Nick Stanger, Margaret Sampson, Mark S. Tremblay

**Affiliations:** 1 Healthy Active Living and Obesity Research Group, Children’s Hospital of Eastern Ontario Research Institute, 401 Smyth Road, Ottawa, ON K1H 8L1, Canada; E-Mails: rlarouche@cheo.on.ca (R.L.); mtremblay@cheo.on.ca (M.S.T.); 2 School of Population & Public Health, University of British Columbia, 2206 East Mall, Vancouver, BC V6H 3V4, Canada; E-Mail: rlgibbons6@gmail.com; 3 Department of Physical Education and Health, Queen Maud University College of Early Childhood Education, Thrond Nergaards Vei 7, NO-7044 Trondheim, Norway; E-Mail: ebs@dmmh.no; 4 Bienenstock Natural Playgrounds, 64 Hatt Street, Dundas, ON L9H 7T6, Canada; E-Mail: adam@naturalplaygrounds.ca; 5 British Columbia Injury Research & Prevention Unit, Child & Family Research Institute, British Columbia Children’s Hospital, University of British Columbia, F511-4480 Oak Street, Vancouver, BC V6H 3V4, Canada; E-Mail: mbrussoni@cw.bc.ca; 6 Department of Pediatrics, School of Population & Public Health, British Columbia Children’s Hospital, University of British Columbia, F511-4480 Oak Street, Vancouver, BC V6H 3V4, Canada; 7 Evaluation Platform on Obesity Prevention, Quebec Heart and Lung Research Institute, Laval University, 2725 Chemin Ste-Foy, Local Y4283, Quebec, QC G1V 4G5, Canada; E-Mail: guylaine.chabot@criucpq.ulaval.ca; 8 School of Architecture and Landscape Architecture, University of British Columbia, 379-2357 Main Mall, Vancouver, BC B6T 1Z4, Canada; E-Mail: susan.herrington@ubc.ca; 9 School of Kinesiology and Health Studies, Queen’s University, 99 University Avenue, Kingston, ON K7L 2P5, Canada; E-Mail: ian.janssen@queensu.ca; 10 Department of Public Health Sciences, Queen’s University, Carruthers Hall, Kingston, ON K7L 2P5, Canada; E-Mail: will.pickett@queensu.ca; 11 Forest School Canada, 411 Corkstown Road, Ottawa, ON K2K 2Y1, Canada; E-Mail: mpower@forestschoolcanada.ca; 12 Department of Environmental Studies, Huxley College of the Environment, Western Washington University, 416 High Street, Bellingham, WA 98225, USA; E-Mail: nick.stanger@wwu.edu; 13 Library Services, Children’s Hospital of Eastern Ontario, 401 Smyth Road, Ottawa, ON K1H 8L1, Canada; E-Mail: msampson@cheo.on.ca; 14 Department of Pediatrics, Faculty of Medicine, University of Ottawa, 401 Smyth Road, Ottawa, ON K1H 8L1, Canada

**Keywords:** outdoor time, physical activity, sedentary behaviour, cardiorespiratory fitness, musculoskeletal fitness, motor skill development, children

## Abstract

The objective of this systematic review was to examine the relationship between outdoor time and: (1) physical activity, (2) cardiorespiratory fitness, (3) musculoskeletal fitness, (4) sedentary behaviour; or (5) motor skill development in children aged 3–12 years. We identified 28 relevant studies that were assessed for quality using the GRADE framework. The systematic review revealed overall positive effects of outdoor time on physical activity, sedentary behaviour, and cardiorespiratory fitness, although causality could not be assumed due to a lack of RCTs. Motor skill development was unrelated to outdoor time; however, this relationship was only examined in a single study of preschool children. No studies were found that examined associations between outdoor time and musculoskeletal fitness.

## 1. Introduction

High levels of physical inactivity and sedentary behaviour are a global concern. Self-reported data from 39 countries indicate that only 23% and 19% of 11 and 13 year olds achieve the recommended 60 min per day of moderate-to-vigorous physical activity (MVPA) [1]. Objectively measured physical activity data show that among Canadian children aged 6- to 10-years, 14% of boys and 7% of girls achieve the recommendations, and they spend an average of 7.4 h per day being sedentary [2]. Data from 27 countries point toward worldwide declines in the performance of children in the 20 m shuttle run test over the last few decades [3].

In parallel with these trends, evidence suggests that the current generation of children play outside less frequently and for shorter durations than their parents’ generation did [4,5]. It has been suggested that children’s physical activity is moving away from unstructured and unsupervised outdoor play toward structured and supervised activities that primarily occur indoors [6,7]. Certain parenting practices common among the middle-class are partially responsible for this shift. The prioritization of academic achievement [8,9] and the tendency for middle-class parents to engage in “concerted cultivation”—Enrolling children in an abundance of extra-curricular enrichment activities, has left children with little unstructured time [8,10]. Heightened concerns for child safety (*i.e.*, injuries, strangers, gangs, and other hazards) [5,6,11,12,13] may also be among the forces pushing children indoors. Although some children have said they prefer to play outside when given the choice [14,15,16], others have reported that they are drawn indoors by interest in sedentary activities such as screen time (e.g., watching TV, playing video games, using the internet), listening to music, art, and reading [17]. This is likely motivated, in part, by the changing nature of children’s social environments, which occurs decreasingly outside and increasingly on screens [18].

Several researchers have proposed that increasing outdoor time could be an effective strategy for limiting sedentary behaviour and increasing physical activity and fitness in children e.g., [19,20]. However, researchers typically cite only a few cross-sectional studies, if any, as evidence of a relationship with outdoor time. Recently published reviews have identified outdoor time as one of several correlates of children’s physical activity behaviour [21,22,23,24]. However, none of these reviews goes beyond the identification of cross-sectional relationships, which typically provide insufficient evidence of a causal relationship between outdoor time and physical activity [21,22,23,24]. Relationships between outdoor time and children’s physical fitness and sedentary behaviour have not been examined systematically to our knowledge. Therefore, the purpose of the current study was to systematically review and evaluate the evidence on the relationship between outdoor time and: (1) physical activity, (2) cardiorespiratory fitness, (3) musculoskeletal fitness, (4) sedentary behavior; and (5) motor skill development in children aged 3–12 years. Our review aimed to include prospective and experimental studies in addition to cross-sectional studies.

## 2. Methods

This systematic review is registered with the international prospective register of systematic reviews PROSPERO network (registration No. CRD42014009307), and followed the PRISMA statement for reporting systematic reviews [25].

### 2.1. Study Inclusion Criteria

We aimed to identify all studies that examined the relationship between outdoor time and physical activity, sedentary behaviour, fitness, or motor skill development in children. Study designs eligible for inclusion were randomized controlled trial (RCT) and non-randomized controlled study (NRS) designs (e.g., cross-sectional, retrospective cohorts, prospective cohorts, case-control). Longitudinal studies were only included if there was an assessment of outdoor time within our set age limits. Studies in languages other than English were included if we were able to translate results using Google Translate.

Study inclusion was dependent upon meeting the pre-determined population, intervention, comparator, and outcome (PICO) study criteria [26]. *Population:* Apparently healthy children (*i.e.*, no clinical diagnosis) aged 3.00–12.99 years. If age was not reported but school year/grade was, the standard regional age range associated with that year/grade was used to determine eligibility for inclusion. *Intervention (exposure)*: Duration of outdoor time. *Comparator*: Indoor time or various durations of outdoor time. Eligible contexts included outdoor settings in general and those that contrasted outdoor and indoor time under specific circumstances (e.g., an unstructured school recess held indoors compared to outdoors). Eligible measures of outdoor time and indoor time included objective measures (e.g., global positioning system), direct observation (e.g., momentary time sampling), and subjective assessments (e.g., proxy- and self-reported outdoor time). *Outcome:* Five movement behaviours and aspects of physical fitness were chosen as outcome indicators by expert agreement. The five eligible indicators in this review were:

1. Physical activity (any intensity and all forms)
2. Cardiorespiratory fitness (submaximal exercise capacity, maximal aerobic power, heart functions, lung functions, blood pressure)
3. Musculoskeletal fitness (power, strength, endurance, bone density)
4. Sedentary behaviour (prolonged sitting, screen time such as watching television, playing video games, computer use, or motorized transportation)
5. Motor skill (agility, balance, coordination, speed of movement)

Physical activity and sedentary behaviour were categorized depending on whether they were assessed as acute or habitual outcome behaviours. To be categorized as acute (single bout), the outcome behaviour must have been reported separately during outdoor time and during indoor time, such that it was possible to compare the behaviour in outdoor and indoor settings. For example, a study could have compared the proportion of time spent sedentary when children were outdoors *vs.* when they were indoors. To be categorized as habitual (typical/usual), the assessment of indoor/outdoor time and the outcome needed to be reported in generalities, such that it was possible to conclude that children who typically spend a certain amount of time outdoors accumulate a certain overall level of the outcome behaviour. For example, the typical duration spent outside each day and weekly physical activity.

### 2.2. Search Strategy

The electronic search strategy was created by M.S. The following databases were searched using the Ovid interface: MEDLINE including In-Process & Other Non-Indexed Citations (1946 to 31 March 2014), Embase (1980 to 2014 week 3), and PsycInfo (1806 to March week 4 2014). CINAHL and SportDiscus were searched using the EBSCOhost interface (database dates not given). No language or study design limits were applied. The search strategies are presented in Supplementary File 1. All co-authors were asked to recommend potentially relevant studies to assist M.S. in the development of the search strategy. Key content experts (*n* = 5) were asked to scan their personal libraries to identify the most influential papers on outdoor time, physical activity, sedentary behaviour, and physical fitness related outcomes in children. No new relevant articles were identified through key content experts, suggesting it is unlikely key articles were missed by the search.

References were imported into Reference Manager Software (Thompson Reuters, San Francisco, CA, USA) where duplicate references were removed. Two independent reviewers screened titles and abstracts of potentially relevant articles, and subsequently examined all potentially relevant full text articles (Rebecca Gibbons and Richard Larouche). Disagreements were resolved by discussion and consensus between the two reviewers or by a third reviewer (Casey Gray) if consensus could not be achieved.

## 2.3. Data Extraction and Quality Assessment

Important features of each study (*i.e.*, year, study design, country, number of participants, age, results, risk of bias, consistency of results, directness of the intervention, precision of results, and evidence of a dose-response gradient) were extracted and recorded in Microsoft Excel by Rebecca Gibbons and checked by Casey Gray The quality of evidence for all studies was assessed by Casey Gray and a subset was checked by Richard Larouche. The Cochrane Handbook (http://handbook.cochrane.org/) was used to evaluate the risk of bias for each individual study in this review. The Grading of Recommendations Assessment, Development and Evaluation (GRADE) framework was used to evaluate the overall quality of the evidence from this systematic review [27].

## 2.4. Analysis

Relationships between outdoor time and the outcomes were categorized in the results according to whether they were examined as “acute” or “habitual” outdoor time behaviours or fitness outcomes. Meta-analyses were planned if the data could be meaningfully pooled (*i.e.*, if data were homogenous in terms of statistical, clinical, and methodological characteristics). Where meta-analyses were not possible, narrative syntheses structured around the type of outcome were conducted. Examination of study findings by sex and age subgroups was planned if sufficient data were available.

## 3. Results

**Figure 1:**
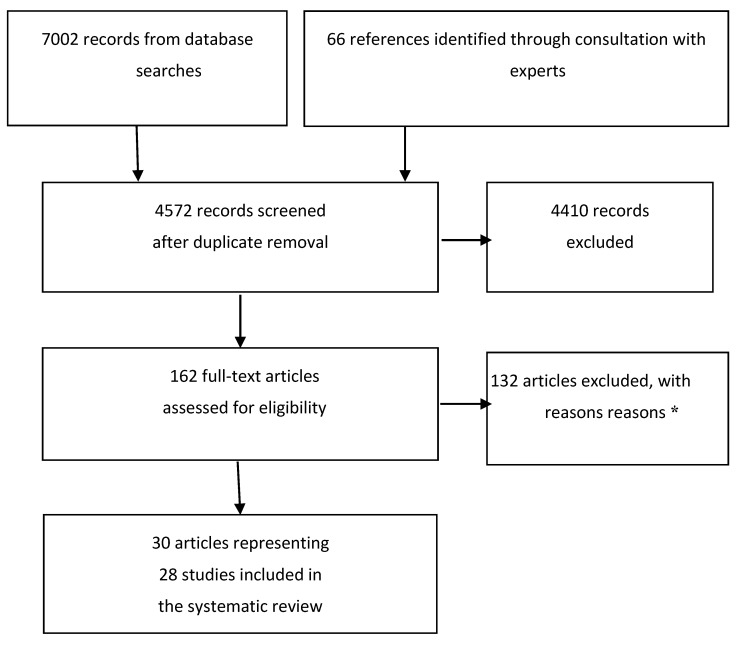
PRISMA flow diagram. * Reasons for exclusions included ineligible exposure (n = 37), ineligible comparator (n = 28), ineligible outcome (n = 39), ineligible population (n = 1), other (includes non-relevant content, review, conference abstract, editorial, incorrect reference (n = 27). Many studies were excluded for multiple reasons. Adapted from Moher, D., *et al*. [25].

The PRISMA flow diagram for study inclusion and exclusion is provided in Figure 1. All studies included in the review are summarized in Table S1 and all studies captured by our search are included in Supplementary Files 2–4. Twenty-eight eligible studies (30 papers; 28 cross-sectional, two longitudinal) from nine countries were identified, with a cumulative sample of 13,798 participants. Most studies reported physical activity as an outcome. Several studies also included at least one other behaviour or fitness outcome. Due to heterogeneity in study design and measures of outdoor time, physical activity, and sedentary behaviour, meta-analyses were not possible. Three of the included papers described apparent differences in physical activity levels for indoor and outdoor time but did not assess statistical significance and were retained for descriptive purposes. Narrative syntheses were conducted for all included studies. Quality of evidence is provided in the Summary of Findings in Table 1, Table 2 , Table 3, Table 4, Table 5 and Table 6.

### 3.1. Studies Based on Inferential Statistics

#### 3.1.1. Habitual Physical Activity

Sixteen observational studies (one longitudinal, 15 cross-sectional) examined the relationship between usual outdoor time and habitual physical activity in 17 papers. All 16 studies reported that outdoor time was positively related to physical activity. Physical activity was assessed in a variety of ways in these studies including pedometer steps counts [28,29], accelerometer readings of total physical activity [30,31] and MVPA [31,32,33,34,35,36], parent- and self-reported leisure time physical activity [37,38], and observed total physical activity and MVPA [39,40,41,42,43,44]. The results were consistent across sexes and different age groups. Only one study showed that outdoor time was not related to physical activity for at least one segment of the sample. Although they showed that outdoor time was related to physical activity levels for older children, there were no associations reported for younger (5- to 6-year-olds) children, or for older (10- to 12-year-olds) girls at particular time points (weekends during warmer months at time 1, or at time 2) [33].

**Table 1:** Associations between outdoor time and habitual physical activity in children.

Quality Assessment							No. of Participants	Absolute Effect (95% CI, SE)	Quality
No. of Studies	Design	Risk of Bias	Inconsistency	Indirectness	Imprecision	Other			
Age range between 29 months and 16 years, data collection occurred over 1 h up to 1 year, physical activity was measured by self-report, parent-report, direct observation, pedometry, and accelerometry									
16	NRS **^a^**	Serious risk of bias **^b^**	No serious inconsistency **^c^**	No serious indirectness **^d^**	Serious imprecision **^e^**	None	8305	Due to heterogeneity in study design and measures of outdoor time, physical activity, and sedentary behaviour, meta-analyses were not possible.	LOW

Notes: GPS, Global Positioning System; h/week, hours per week; ICC, intra-class correlation; inconsistency = heterogeneity; indirectness = substantial differences exist between the population, the intervention, or the outcomes under consideration in the review; imprecision = random error; MVPA, moderate-to-vigorous physical activity; NRS, non-randomised study; PA, physical activity; Risk of Bias = internal validity; T1, time 1; T2, time 2. 16 non-randomised studies in 17 papers [28,29,30,31,32,33,34,35,36,37,38,39,40,41,42,43,44]. **^a^** Includes 15 cross-sectional studies [28,29,30,31,32,34,35,36,37,38,39,40,41,42,44] and 1 longitudinal study [33]. Note: McKenzie[43] and Sallis [44] are publications from the same study. **^b^** Unknown reliability of the proxy measures of outdoor play. Unknown accelerometer cut-points. Analyses did not control for important potential confounders (*i.e.*, age and gender) [30]. Test-retest reliability of the questions on outdoor play was only fair to moderate (ICC 0.21–0.46) [33]. Unknown validity and reliability of the physical activity measure, or the allocation of PA into low, medium and high status [37]. No validity or reliability information on indoor/outdoor time parent report questionnaire [31]. Statistically significant differences between included/excluded participants. Validity and reliability of the measure of outdoor play are unknown [34]. Large amount of missing data that likely leads to underestimation of PA time. *i.e.*, the GPS were presumed to be switched off for 33.7% of activity bouts, which activity diaries showed often occurred because children participating in organised sports were requested to remove the monitors [35]. Unknown if outcome measures were completed, unknown if all measured outcomes were reported. No details about outdoor measures or PA measures [41]. Little information on outdoor measure, unknown validity, reliability [29]. Positive associations between outdoor time and PA were reported in the abstract, stating that outdoor time explained 19% of the variance in PA and that time spent outside and social support derived from friends were the strongest predictors examined of PA examined in their study. Regression table of reported contribution of outdoor time to PA is missing from the paper [38]. No information about outdoor measure, unknown validity and reliability [32]. No information about survey items or psychometrics provided for time use survey [36]. **^c^** *On weekends*: At T1 there were no cross-sectional associations between time outdoors and MVPA during warmer months, for younger children during cooler months, or prospectively; At T2 outdoor time was not related to MVPA during warmer or cooler months for younger boys, younger girls, or older girls. *On weekends*: There were no relationships between outdoor time and MVPA for younger boys or girls; no prospective relationships for older girls or younger children; At T2 there were no cross-sectional associations for older girls or younger children [33]. **^d^** Step counts were assessed as a surrogate measure of PA. Therefore intensity of PA was not assessed [28]. **^e^** The magnitude of the median sample size was intermediate. The magnitude of the number of included studies was high.

**Table 2:** Associations between outdoor time and acute physical activity.

Quality Assessment									No. of Participants	Absolute Effect (95% CI, SE)	Quality
No. of Studies		Design		Risk of Bias	Inconsistency	Indirectness	Imprecision	Other			
Age range between 3 and 16 years, data collected over ~45 min up to 1 school year, acute physical activity was assessed by pedometer, direct observation, parent report, self-report											
8	NRS **^a^**		No serious risk of bias **^b^**		No serious inconsistency **^c^**	No serious indirectness **^d^**	No serious imprecision **^e^**	None	3522	Due to heterogeneity in study design and measures of outdoor time, physical activity, and sedentary behaviour, meta-analyses were not possible.	MODERATE

Notes: x̄, mean; Inconsistency = heterogeneity; indirectness = substantial differences exist between the population, the intervention, or the outcomes under consideration in the review; imprecision = random error; MVPA, moderate-to-vigorous physical activity; NRS, non-randomised study; PA, physical activity; Risk of Bias = internal validity; SD, standard deviation; y/o, years old. 8 non-randomised studies [45,46,47,48,49,50,51,52]. **^a^** Includes 7 cross-sectional [45,46,47,48,50,51,52] and 1 longitudinal study [49]. **^b^** More adolescents (11–13 y/o) and students from 2 schools dropped out after providing informed consent (*p* < 0.001). There was a significant difference between those who provided complete measures and those excluded (*n* = 353) by school and age (*p* < 0.001) [47]; Teachers were instructed to behave normally while their classrooms were being observed but it is possible they made adjustments to their classes during observations [50]. **^c^** There was not a significant difference for the % of class time that was very active outdoors (x̄ = 14.83, SD = 8.04) *vs.* indoors (x̄ = 13.96, SD =7.46). There was not a significant mean difference in minutes of PA for standing, walking, being very active, or engaging in MVPA during an indoor *vs.* outdoor lesson [50]. Preschool girls’ MVPA not significantly different indoors from outdoors [51]. **^d^** Outcome timeframe was likely insufficient for 1 study that collected accelerometry data for 1 preschool day, as participants were not provided with a “period of habituation” to the device [51]. **^e^** The magnitude of the median sample size was intermediate (N = 170). The magnitude of the number of included studies was moderate (N = 8).

**Table 3:** Associations between outdoor time and habitual sedentary behaviour.

Quality Assessment								No. of Participants	Absolute Effect (95% CI, SE)	Quality
No. of Studies	Design		Risk of Bias	Inconsistency	Indirectness	Imprecision	Other			
Age range between 3 and 10 years, data collected over 1 day up to 1 week, sedentary time measured using direct observation and accelerometry										
2	NRS **^a^**	Serious risk of bias **^b^**		No serious inconsistency	No serious indirectness **^c^**	Serious imprecision **^d^**	None	437	% difference = −4.4; CI: −7.3–−1.4, *p* = 0.005 **^e^**	LOW

Note: x̄, mean; inconsistency = heterogeneity; indirectness = substantial differences exist between the population, the intervention, or the outcomes under consideration in the review; imprecision = random error; NRS, non-randomised study; risk of bias = internal validity; 2 non-randomised study [34]. **^a^** Includes 2 cross-sectional studies [34]. **^b^** There is a risk of bias due to selective reporting. Jones [35] indicated in their methods that sedentary bouts were collected but did not report results. No information was provided about the outdoor variable re: Validity, reliability, or wording of item [34]. **^c^** accelerometers were only worn for 1 day. Participants were not provided with a period of habituation. **^d^** The magnitude of the median sample size was intermediate. The magnitude of the number of included studies was small (N = 2). **^e^** Children’s sedentary time was x̄ = 6.1 (1.3) h/day. Children who spent 1 h or more outdoors had 4.4% less sedentary time than children who spent less than 1 h per day outdoors. (Adjusted for group and hours of monitoring) [34].

**Table 4:** Associations between outdoor time and acute sedentary behaviour.

Quality Assessment							No. of Participants	Absolute Effect (95% CI, SE)	Quality
No. of Studies	Design	Risk of Bias	Inconsistency	Indirectness	Imprecision	Other			
Age range between 3 and 10 years, data collected over 1 day up to 1 week, sedentary time measured using direct observation and accelerometry.									
3	NRS **^a^**	Serious risk of bias **^b^**	No serious inconsistency	No serious indirectness **^c^**	Serious imprecision **^d^**	None	925	Due to heterogeneity in study design and measures of outdoor time, physical activity, and sedentary behaviour, meta-analyses were not possible.	LOW

Notes: inconsistency = heterogeneity; indirectness = substantial differences exist between the population, the intervention, or the outcomes under consideration in the review; imprecision = random error; NRS, non-randomised study; risk of bias = internal validity. 3 non randomised studies [48,50,51]. **^a^** Includes 3 cross-sectional studies [48,50,51]. **^b^** When assessing outdoor location and sedentary time by direct observation, the authors noted that, although teachers were instructed to behave normally while their classrooms were being observed, they may have made changes to their classes while they were being observed [50]. **^c^** accelerometers were only worn for 1 day. Participants were not provided with a period of habituation [51]. **^d^** The magnitude of the median sample size was small. The magnitude of the number of included studies was small (N = 3).

**Table 5:** Associations between outdoor time and motor skills.

Quality Assessment							No. of Participants	Absolute Effect (95% CI, SE)	Quality
No. of Studies	Design	Risk of Bias	Inconsistency	Indirectness	Imprecision	Other			
Age ranged between 3 and 4 years, data collected at 1 time point, motor skill measured by the APM Inventory for assessing pre-school children’s perceptual and basic motor skills.									
1	NRS **^a^**	Serious risk of bias **^b^**	No serious inconsistency **^c^**	Serious indirectness **^d^**	Serious imprecision **^e^**	None	105	r = −0.29, *p* = 0.42 **^f^**	VERY LOW

Notes: Inconsistency = heterogeneity; indirectness = substantial differences exist between the population, the intervention, or the outcomes under consideration in the review; imprecision = random error; NRS, non-randomised study; PA, physical activity; risk of bias = internal validity. 1 non randomised study [53]. **^a^** Includes 1 cross-sectional study [53]. **^b^** Subjective measures of motor skills (APM Inventory) and outdoor time [53]. **^c^** Total time playing outdoors was not related to the following motor skills: Walking, standing broad jump, agility, throwing at a target from 2 m or 3 m, throwing-catching combination, clapping, galloping, somersault, kicking a ball [53]. **^d^** Time measured playing outdoors actively and very actively was assessed by parent PA diary. These results were combined to form a “playing outdoors” combined variable preventing a clear comparison of the effects of indoor and outdoor time as separate from the effects of PA [53]. **^e^** The magnitude of the median sample size was intermediate (N = 105). The magnitude of the number of included studies was small (N = 1). **^f^** More time playing outdoors was correlated with faster completion of 10 m run [53].

**Table 6:** Associations between outdoor time and blood pressure.

Quality Assessment							No. of Participants	Absolute effect (95% CI, SE)	Quality
No. of Studies	Design	Risk of Bias	Inconsistency	Indirectness	Imprecision	Other			
Age ranged between 3 and 7 years, data collected over 1 week, BP was assessed by Dinamap and Automated Sphygmomanometer									
2	NRS **^a^**	Serious risk of bias **^b^**	Serious inconsistency **^c^**	Serious indirectness **^d^**	Serious imprecision **^e^**	None	1531	highest vs. lowest tertile of outdoor PA (≥0.57 h/day vs. ≤0.14 h/day) diastolic BP x̄ = 61.5 (59.5–63.5) vs. x̄ = 63.0 (60.8–65.2), *p* = 0.01 highest vs. lowest tertile of indoor PA (≥0.18 h/day vs. ≤0.06 h/day) systolic BP x̄ = 98.9 (97.7–100.1) vs. x̄ = 101.2 (99.6–102.8), *p* = 0.03 indoor PA and elevated BP OR = 0.50; CI: 0.27–0.92, *p* = 0.03 **^f^**	VERY LOW

Notes: x̄, mean; BP, blood pressure; CI, Confidence Interval; inconsistency = heterogeneity; indirectness = substantial differences exist between the population, the intervention, or the outcomes under consideration in the review; imprecision = random error. OR, odds ratio; PA, physical activity; risk of bias = internal validity; 2 non-randomised studies [53,54]. **^a^** Includes 2 cross-sectional studies [53,54]. **^b^** Outdoor time was assessed by subjective measures [53]. **^c^** Systolic BP was positively correlated with playing outdoors, and was unrelated to playing indoors. Diastolic BP was not correlated with playing indoors or outdoors [53]. **^d^** Time measured playing indoors and outdoors actively and very actively was assessed by parent report. These results were combined to form a “playing indoors” and “playing outdoors” combined variable, preventing a clear comparison of the effects of indoor and outdoor time as distinguished from PA [53]. Time measured in identified activities and location of each activity was assessed for hours/week parent-report, preventing a clear comparison of the effects of indoor and outdoor time as distinguished from PA, on BP levels [54]. **^e^** The magnitude of the median sample size was small. The magnitude of the number of included studies was small (N = 2). **^f^** Children in the highest tertile of outdoor activity had a significantly lower diastolic and systolic BP than children in the lowest tertile of outdoor activity. Indoor activities were associated with an increased likelihood of having an elevated BP [54].

Seven studies reported correlations between outdoor time and habitual physical activity [29,30,31,38,41,43,44]. They reported values ranging from 0.20 to 0.74, implying that 4% to 55% of the variance in physical activity was accounted for by outdoor time. The amount of explained variance increased when outdoor time was included in models that also examined other correlates of physical activity, accounting for 25% to 75% of the variance in physical activity (Median R^2^ = 0.599) [39,40,42]. Investigating outdoor time by season [32] showed that higher amounts of outdoor time increased the likelihood of achieving higher amounts of MVPA by 1.3% to 12.4% in both summer and winter months [32]. Cleland *et al.* showed that each additional hour per week spent outdoors during the cooler months was associated with an extra 26.5 min per week of MVPA among older girls, and 21 min per week of MVPA among older boys [33]. Longitudinally, each additional hour spent outdoors on weekends at baseline was associated with an extra 5 min per week MVPA among older girls and boys 3 years after baseline [33].

#### 3.1.2. Acute Physical Activity

Eight observational studies (one longitudinal, seven cross-sectional) examined the relationship between outdoor time and acute physical activity. All eight studies reported that physical activity was higher when children were outdoors than when they were indoors and five studies showed that total physical activity was 2.2 to 3.3 times higher outdoors than indoors [45,46,48,51]. Physical activity was assessed in a variety of ways in these studies including pedometer step counts [49], accelerometer readings of total physical activity [45,46,48,52] and MVPA [46,47,51,52], and direct observation [50]. The results were consistent across sexes, different age groups, and contexts, *i.e.*, afterschool [46,52], weekends [46,52], at school/preschool [49,50,51]. Only Skala *et al.* showed that physical activity was not higher outdoors than indoors for one observed intensity level [50]. Although they showed that children spent more time standing, walking, and engaging in MVPA in physical education classes held outdoors compared with indoors, there was no difference for observed time being “very active” [50].

#### 3.1.3. Habitual Sedentary Behaviour

One cross-sectional study examined the relationship between outdoor time and acute sedentary behaviour [34]. This study showed that outdoor time was negatively related to minutes per day of total sedentary time as measured by accelerometers [34]. Children who spent 1 h or more outdoors had 4.4% less sedentary time than children who spent less than 1 h per day outdoors (adjusted for study site cluster and hours of monitoring) [34].

#### 3.1.4. Acute Sedentary Behaviour

Three observational studies (all cross-sectional) examined the relationship between outdoor time and acute sedentary behaviour. All three studies showed that sedentary time was lower while children were outdoors than when they were indoors. Sedentary behaviour was assessed by direct observation [50] and accelerometers [48,51]. The results were consistent in preschool [48,51] and physical education [50] settings. Preschoolers spent up to twice as much time being sedentary while indoors than they did while outdoors [51] and spent a mean difference of 14% more time being sedentary while indoors [48]. During physical education classes, Skala [50] observed that children had a mean difference of 10% more time sitting in indoor physical education classes compared with outdoor classes.

#### 3.1.5. Motor Skills

One cross-sectional study examined the relationship between outdoor time and fundamental motor skills. Motor skills were assessed by the APM-inventory [55]. This study showed that parent-reported time playing outdoors was correlated with faster completion of a 10 metre run (r = −0.29, *p* = 0.42), however no other motor skills (walking, standing broad jump, agility, throwing at a target from 2 or 3 m, throwing-catching combination, clapping, galloping, somersault, kicking a ball) significantly differed by indoor or outdoor location [53].

#### 3.1.6. Cardiorespiratory Fitness

Two cross-sectional studies investigated the relationship between outdoor time and blood pressure. Arterial blood pressure was assessed in each study by automated sphygmomanometers (HEM 907; Omron Healthcare, Bannockburn, IL, USA) [54] or Dinamap (Critikon model 1846, Tampa, FL, USA) [53]. There was no clear pattern of results. Outdoor activity was related to lower diastolic blood pressure in one study [54] and higher systolic blood pressure in the other study [53]. Outdoor time was not related to other measures of blood pressure in Saakslahti’s [53] study. Indoor activity was related to lower systolic blood pressure, diastolic blood pressure, and mean arterial blood pressure [54].

### 3.2. Descriptive Studies

Three studies measured eligible study exposures and outcomes but did not assess statistical significance, and are reported here for discussion purposes. All three studies discussed higher physical activity and lower sedentary behaviour when preschoolers were outdoors compared with indoors [56,57,58]. It should be noted that the same sample described by Durant [57] was examined by Baranowski *et al.* [39].

### 3.3. Summary of Findings

Twenty-eight observational studies met the inclusion criteria. Few studies examined cardiorespiratory fitness and no studies investigated musculoskeletal fitness. Overall, time spent outdoors was consistently associated with improved movement behaviour (*i.e.*, more physical activity and less sedentary behaviour) and fitness outcomes when compared with time spent indoors. The low risk of bias of included studies, the consistency of findings, and large effect sizes prompted us to upgrade the Quality of Evidence rating from “low” to “moderate” for the acute physical activity outcome. The small number of observational studies and the lack of clarity around the relationships in studies that examined motor skill and blood pressure in relation to outdoor time warranted downgrading the Quality of Evidence for these outcomes from “low” to “very low”.

## 4. Discussion

This review found that outdoor time is positively related to physical activity and negatively related to sedentary behaviour in children aged 3–12 years. Studies that examined habitual behaviours showed that children with higher amounts of outdoor time engaged in higher amounts of physical activity and lower amounts of sedentary behaviour than children who spend less time outdoors. Studies that examined acute behaviours showed that children were more physically active and less sedentary while they were outside than while they were inside.

All of the studies examined showed that physical activity was higher outdoors compared with indoors. The consistent positive relationship between outdoor time and physical activity held across sexes, age groups (preschoolers, school-aged children), and contexts (full day, leisure time, adult directed). Our finding that outdoor time is a positive correlate of physical activity is consistent with three previous systematic reviews and one review of reviews carried out with preschoolers, children and youth [22,23,24,59]. Between them, the four reviews captured a total of four studies in 6 papers [28,39,40,43,44,57], all of which were also captured by our search, increasing our confidence that our review was comprehensive. Of the studies included, all reported small sample sizes (N’s = 191–246) and relied on direct observation of outdoor time; three studies used direct observation to assess physical activity and one used pedometers. Furthermore, the previous reviews were designed to examine correlates of physical activity, and did not assess the risk of bias or overall quality of evidence. We found that all of the studies included in our review were also observational and assessed exposures and outcomes of interest simultaneously rather than prospectively, which may limit our ability to infer causal relationships between outdoor time and identified outcomes. The design of included studies also prevented us from assigning a high quality of evidence rating in accordance with GRADE framework. However, the use of objective measures of physical activity and sedentary behaviour (e.g., accelerometers) and outdoor location (*i.e.*, GPS) in some studies, and the consistency of the results pertaining to the relationships between outdoor time, physical activity and sedentary behaviour is reason not to rule out the possibility of causation.

Four studies examined sedentary behaviour in this review. Studies conducted in preschool settings showed that increased time outside at preschool was related to lower sedentary behaviour [34,48,50,51]. Interestingly, this contrasts with the findings of Dowda and colleagues [60], who identified that preschool policies promoting outdoor time had no effect on preschoolers’ sedentary behaviour levels. Rather than concluding outdoor time did not influence sedentary behaviour, it may be that policies to increase outdoor time in early childcare centres were not implemented appropriately [60], that outdoor play policies hindered movement, or that the outdoor play spaces were not challenging enough [61].

To the best of our knowledge, this is the first attempt to systematically review the literature on the effects of outdoor time on cardiorespiratory fitness, musculoskeletal fitness, or motor skill development in children. Very few studies that examined indicators of cardiorespiratory fitness and motor skill development were identified in our review, and those that did reported no clear relationship with outdoor time. Schaefer and colleagues [62] reported positive cross-sectional relationships between self-reported time spent outdoors after school and Leger shuttle run scores in 9–17 year olds (x = 13.6 ± 1.4 years), with children who spend most or all of their afterschool time outdoors achieving higher cardiorespiratory fitness than children who spend none of their after school time outside. No differences were found for outdoor time and blood pressure [62]. This study was ineligible for our review because the mean age of participants was outside of our target range; however, these results suggest that outdoor time may be associated with greater aerobic fitness in children and youth similarly across age ranges.

This review examined outdoor *versus* indoor time in general. It did not assess outcomes in relation to the quality of the outdoor environment, which has been suggested to be an important correlate of physical activity [63]. For example, it is plausible that greater benefits would be derived from more natural outdoor settings. Proximity to undeveloped green space (e.g., accessible wooded areas) was related to increased physical activity in a sample of 11–13 year old participants of the Canadian Health Behaviour in School-Aged Children survey [64]. Positive relationships in increasingly natural settings may be particularly beneficial for blood pressure, as adult studies have shown that outdoor exercise lowers systolic blood pressure more than treadmill training [57]. Other environmental characteristics have also been forwarded as promoting physical activity or reducing sedentary behaviour in outdoor environments. For instance, McKenzie [42] observed that children more frequently prompt each other to be active outdoors than indoors, and prompt each other to be sedentary more often indoors than outdoors. Parents are also very likely to prompt children to be sedentary while indoors. Alternatively, increasingly natural environments may not improve the relationship between outdoor time and movement behaviours universally. For example, children who reside in neighbourhoods marked by high levels of physical disorder and those living in public housing played outdoors more often than other children [65]. There is a need for future research to further elucidate the role of nature in the relationships between outdoor time and movement behaviours. A combination of accelerometers with GPS and geographic information systems can be used to provide greater insights into the characteristics of outdoor environments that promote or discourage physical activity [66].

Increasing the amount of time children spend outdoors has been recommended as a promising strategy for increasing children’s physical activity levels [19,20]. Child reported preferences for outdoor play suggest interventions to increase outdoor time would be well-received by the targeted population [14,15]. However, some public health campaigns have collectively recommended keeping children indoors for almost the entire day. These include campaigns that recommend staying indoors during peak daylight hours (10:00 am–4:00 pm) to avoid sun exposure and melanoma risk (e.g., http://www.healthlinkbc.ca/healthfiles/pdf/hfile26.pdf) [67], campaigns that recommend staying indoors in the rush-hour periods before and after school to avoid traffic related air pollution (e.g., http://www.peelregion.ca/health/cleanairpeel/smog-health.htm) [68], and campaigns that recommend staying indoors in the evening hours (from dusk to dawn) to prevent mosquito carried illnesses (e.g., http://www.healthlinkbc.ca/healthfiles/pdf/hfile88.pdf) [69]. By continuing to demonstrate that outdoor time is also related to positive health outcomes, researchers can contribute to informing a more balanced perspective to health promotion and chronic disease prevention.

Efforts to increase children’s outdoor time face another barrier—Parent concerns about child safety and prioritizing of academic achievement over physical health [3,4,5,6,7]. Some have said that this is having a negative effect on children’s outdoor time [6,7,12]. There is a clear need to convey the serious importance of increasing physical activity and decreasing sedentary behaviour on the present and future health of children and youth, and to understand children’s need to experience increasing levels of challenge such as wandering without adult supervision and other types of “risky play” [70,71].

### Strengths and Limitations

There were several strengths associated with this systematic review. Our comprehensive search strategy was developed by a research librarian with specific expertise in the systematic reviews with guidance from a strong team of content area experts. Our inclusion and exclusion criteria and analysis plan were registered on PROSPERO *a-priori* for improved transparency. We included objective and subjective measures of outdoor time, allowing us to comment on the duration of outdoor time, but also the specific context; four studies used GPS to determine indoor and outdoor location [35,45,47,52], while the remaining studies used direct observation [28,31,39,40,42,43,48,49,50,51,56], proxy-report [29,30,31,32,33,34,37,41,53,54], and self-report [36,38,46,58]. Finally, we followed systematic processes for examining the risk of bias and quality of included studies [27], which enabled the synthesized results to be interpreted within the context of the inherent limitations of each study. The observational nature of included studies automatically increases the risk of bias associated with studies in this review. However, there were few other factors of concern.

Limitations of this review included heterogeneity in the measurement of outdoor time and outcome variables across the included studies. As such, while it was possible to ascertain the direction of the detected relationships, it was not possible to quantify the overall effect size for each outcome. All of the included studies relied on observational designs, and nearly all (*i.e.*, 26 of 28 studies) used cross-sectional designs. There is a clear need for more evidence from studies using randomized research designs to examine whether increasing outdoor time leads to increased physical activity and decreased sedentary time. Without these experimental designs it was difficult to definitively state if there are cause-effect relationships between outdoor time and included outcomes. Finally, our systematic review predominantly included studies from developed nations, which was a limitation of the evidence base. The type and quantity of physical activity and sedentary behaviour differ between developing and developed nations (e.g., active transportation and subsistence chores are generally more common in rural Africa than in developed nations) [72]. Nevertheless, there is no reason to expect the direction of the relationships between outdoor time and physical activity or sedentary behaviour to differ in developing countries.

## 5. Conclusions

Structured indoor achievement oriented activities (e.g., competitive sports, excessive homework, musical instrument training) seem to be replacing children’s outdoor free time [6,7]. Our systematic review, based on “very low” to “moderate” quality evidence, provides consistent evidence that children aged 3–12 years who spend more time outside are more active and less sedentary. All of the included studies reported positive effects on movement behaviours. Positive findings were apparent across ages, sexes and contexts (e.g., preschool, physical education, leisure time). Our findings highlight the importance of preserving time in children’s schedules for unstructured outdoor play and also for incorporating time outdoors within structured contexts like school and childcare as a means of promoting healthy active living.

## Supplementary Files

Supplementary File 1
